# Improving ensiling characteristics by adding lactic acid bacteria modifies in vitro digestibility and methane production of forage-sorghum mixture silage

**DOI:** 10.1038/s41598-021-81505-z

**Published:** 2021-01-21

**Authors:** Chatchai Kaewpila, Pongsatorn Gunun, Piyawit Kesorn, Sayan Subepang, Suwit Thip-uten, Yimin Cai, Suradej Pholsen, Anusorn Cherdthong, Waroon Khota

**Affiliations:** 1 Faculty of Natural Resources, Rajamangala University of Technology Isan, Sakon Nakhon, 47160 Thailand; 2 Faculty of Liberal Arts and Sciences, Sisaket Rajabhat University, Sisaket, 33000 Thailand; 3 Faculty of Agricultural Technology, Sakon Nakhon Rajabhat University, Sakon Nakhon, 47000 Thailand; 4 Japan International Research Center for Agricultural Sciences (JIRCAS), Tsukuba, Ibaraki 305-8686 Japan; 5 Faculty of Agriculture, Khon Kaen University, Khon Kaen, 40002 Thailand

**Keywords:** Microbiology, Zoology

## Abstract

Improving the nutrition of livestock is an important aspect of global food production sustainability. This study verified whether lactic acid bacteria (LAB) inoculant could promote ensiling characteristics, nutritive value, and in vitro enteric methane (CH_4_) mitigation of forage sorghum (FS) mixture silage in attacking malnutrition in Zebu beef cattle. The FS at the soft dough stage, Cavalcade hay (CH), and cassava chip (CC) were obtained. The treatments were designed as a 4 × 2 factorial arrangement in a completely randomized design. Factor A was FS prepared without or with CH, CC, and CH + CC. Factor B was untreated or treated with *Lactobacillus casei* TH14. The results showed that all FS mixture silages preserved well with lower pH values below 4.0 and higher lactic acid contents above 56.4 g/kg dry matter (DM). Adding LAB boosted the lactic acid content of silages. After 24 h and 48 h of in vitro rumen incubation, the CC-treated silage increased in vitro DM digestibility (IVDMD) with increased total gas production and CH_4_ production. The LAB-treated silage increased IVDMD but decreased CH_4_ production. Thus, the addition of *L. casei* TH14 inoculant could improve lactic acid fermentation, in vitro digestibility, and CH_4_ mitigation in the FS mixture silages.

## Introduction

The use of locally available natural resources to improve the nutrition of livestock is an important aspect of global food production sustainability. In ruminant production, feeding partial mixed rations as the sole diet is a strategy to eliminate malnutrition. In tropical countries, sorghum (*Sorghum bicolor*) cultivation generates a major fodder crop^1,2^. Forage sorghum (FS) harvested at the optimal stage of 75 days has 31.0% dry matter (DM) content, consisting of 60.7% neutral detergent fiber (NDF) with 5.1% crude protein (CP)^3^. With concentrate-free diets, FS resulted in insufficient total digestible nutrients and negative nitrogen balance in ruminants^4^. Therefore, FS is ideal for silage production, providing a roughage source. Pholsen et al.^1^ demonstrated FS-mixture silage production by adding two neighboring crops, Cavalcade hay (CH) at 15% inclusion rate and dry cassava chip (CC) at 10% inclusion rate, to improve the nutritive levels, such as CP and metabolizable energy (ME) contents for maintaining animals’ requirements. Taking mixture silages into the account, the beneficial effect of adding unusual components into the silo on ensiling should be ensured to eliminate unpredictable spoilage.

The addition of lactic acid bacteria (LAB) inoculants is known to increase the initial LAB numbers of silages, prohibiting the growth of enterobacteria and clostridia^5^. LAB application was also reported to reduce enteric methane (CH_4_) production, a major source of energy loss and contributor to ruminants’ greenhouse gas emissions, because it could be a rumen fermentation enhancer^6,7^. When silage is prepared from tropical forages, improved silage quality was verified with *Lactobacillus casei* TH14 inoculant isolated from corn silage^8,9^.

The comparison of silage quality with animal requirements has practical relevance to ensuring livestock performance. This paper hypothesizes that alternative ingredients and LAB additive might alter ensiling characteristics and in vitro ruminal utilization of FS mixture silage, leading to enhanced Zebu beef cattle by improving indigenous materials. Therefore, the purpose of this study was to evaluate the ensiling characteristics, nutritive value, and in vitro CH_4_ production of FS mixture silages (FS, FS + CH, FS + CC, and FS + CH + CC) prepared without and with strain TH14.

## Results

### Microbial population and chemical composition of ensiling material

Microbial counts were reported in colony-forming units (cfu)/g in fresh matter (FM; Table 1). All ensiling materials showed low LAB counts (ranging from 10^3^ to 10^4^) but high counts of aerobic bacteria (10^8^) and yeast (10^6^ to 10^8^). The coliform bacteria were found only in FS (10^7^). The molds were below detection level (< 10^2^). The DM content of FS was 25.97%, whereas those of CH and CC were greater than 88.73%. The CP, NDF, acid detergent fiber (ADF), acid detergent lignin (ADL), and nonfiber carbohydrates (NFC) contents of FS were in between those of CC and CH. The gross energy (GE) contents ranged from 16.22 to 18.00 MJ/kg DM.

**Table 1:** Microbial counts, chemical composition and gross energy content of the ensiling materials used in this study.

Item	FS	CH	CC
**Microbial counts (colony forming unit/g fresh matter)**			
Lactic acid bacteria	5.0 × 10^3^	1.9 × 10^4^	5.0 × 10^3^
Coliform bacteria	5.2 × 10^7^	ND	ND
Aerobic bacteria	1.4 × 10^8^	4.2 × 10^8^	1.9 × 10^8^
Yeast	6.8 × 10^7^	1.7 × 10^8^	2.0 × 10^6^
Mold	ND	ND	ND
**Chemical composition (% dry matter)**			
Dry matter (%)	25.97	96.45	88.73
Organic matter	97.18	93.37	94.43
Crude protein	5.21	15.60	2.21
Ether extract	1.56	1.47	0.49
Neutral detergent fiber	60.42	64.79	19.69
Acid detergent fiber	38.36	50.32	10.56
Acid detergent lignin	4.61	10.07	2.06
Nonfiber carbohydrates	22.99	11.51	72.04
Gross energy (MJ/kg dry matter)	18.00	18.00	16.22

*FS* forage sorghum, *CH* Cavalcade hay, *CC* cassava chip, *ND* not detected.

### Fermentation quality of silage

The highest DM content (p < 0.05) was FS + CH + CC silage, followed by FS + CH (Table 2). The highest pH value was found in either FS + CH silage (p < 0.05) or LAB-untreated (control) silage (p < 0.05). The lactic acid contents did not differ (p = 0.45) among materials but increased (p < 0.05) in LAB-treated silage. The butyric acid content of FS silage was greater (p < 0.05) than that of FS + CH + CC silage. The LAB inoculation tended to affect (p = 0.064) the butyric acid content of silage. The interaction effect (materials × LAB inoculation) was detected (p < 0.05) in the acetic acid, propionic acid, and ammonia nitrogen (NH_3_-N) concentrations.

**Table 2:** Fermentation characteristics of the FS mixture silages.

Item	Dry matter (%)	pH	Fermentation products (g/kg dry matter)				
			Lactic acid	Acetic acid	Propionic acid	Butyric acid	Ammonia nitrogen
FS	24.87^d^	3.72^b^	59.60^b^	24.17^a^	0.77^c^	0.96^a^	1.55^a^
FS + TH14	26.09^cd^	3.65^b^	71.24^ab^	4.49^de^	0.75^c^	0.64^ab^	1.12^b^
FS + CH	34.25^b^	3.93^a^	57.75^b^	17.59^b^	0.74^c^	0.67^ab^	0.71^c^
FS + CH + TH14	33.63^b^	3.81^ab^	71.90^ab^	5.66^d^	0.74^c^	0.54^b^	0.69^c^
FS + CC	29.38^c^	3.78^b^	59.51^b^	17.72^b^	0.73^c^	0.71^ab^	0.71^c^
FS + CC + TH14	28.26^cd^	3.70^b^	87.04^a^	2.91^e^	0.82^c^	0.53^b^	0.85^c^
FS + CH + CC	40.08^a^	3.76^b^	56.40^b^	11.85^c^	1.15^b^	0.42^b^	0.60^c^
FS + CH + CC + TH14	39.16^a^	3.67^b^	87.49^a^	3.77^de^	1.35^a^	0.53^b^	0.64^c^
SEM	1.586	0.048	7.418	0.922	0.038	0.108	0.097
**Material means**							
FS	25.48^d^	3.68^b^	65.42	14.33^a^	0.76^b^	0.80^a^	1.34^a^
FS + CH	33.94^b^	3.87^a^	64.83	11.63^b^	0.74^b^	0.61^ab^	0.70^b^
FS + CC	28.82^c^	3.74^b^	73.27	10.32^b^	0.78^b^	0.62^ab^	0.79^b^
FS + CH + CC	39.62^a^	3.71^b^	71.95	7.81^c^	1.25^a^	0.47^b^	0.62^b^
**Inoculation means**							
Without TH14	32.15	3.80^a^	58.32^b^	17.83^a^	0.85^b^	0.69^a^	0.89
With TH14	31.78	3.71^b^	79.42^a^	4.21^b^	0.92^a^	0.56^b^	0.83
**P value**							
Materials (M)	< 0.001	0.007	0.453	< 0.001	< 0.001	0.023	< 0.001
Inoculation (I)	0.715	0.017	< 0.001	< 0.001	0.023	0.064	0.277
Interaction (M × I)	0.823	0.942	0.368	< 0.001	0.040	0.182	0.018

*FS* forage sorghum, *CH* Cavalcade hay, *CC* cassava chip, *TH14 L. casei* TH14 inoculant, *SEM* standard error of the means.

Means within columns with difference superscript letters differ at p < 0.05.

### Microbial population of silage

The LAB and aerobic bacteria counts of silage ranged from 10^7^ to 10^8^ cfu/g FM (Table 3). The FS + CH + CC silage showed lower (p < 0.05) yeasts than FS + CH, or FS + CC silage did. Moreover, LAB-treated silage decreased (p < 0.05) the yeast counts. In all silages, the coliform bacteria and molds were below detection level (< 10^2^ cfu/g FM). The LAB and aerobic bacteria counts were affected (p < 0.05) by interaction.

**Table 3:** Microbial counts of the FS mixture silages.

Item	Microbial counts (colony forming unit/g fresh matter)				
	Lactic acid bacteria	Coliform bacteria	Aerobic bacteria	Yeast	Mold
FS	1.9 × 10^7d^	ND	2.4 × 10^7b^	2.1 × 10^7bc^	ND
FS + TH14	4.1 × 10^8b^	ND	1.9 × 10^7b^	7.8 × 10^6cd^	ND
FS + CH	2.2 × 10^8c^	ND	2.6 × 10^7b^	2.9 × 10^7ab^	ND
FS + CH + TH14	6.2 × 10^8a^	ND	1.8 × 10^7b^	1.9 × 10^7bc^	ND
FS + CC	1.9 × 10^7d^	ND	3.1 × 10^7b^	4.0 × 10^7a^	ND
FS + CC + TH14	3.9 × 10^8b^	ND	2.2 × 10^7b^	7.9 × 10^6cd^	ND
FS + CH + CC	2.8 × 10^7d^	ND	1.9 × 10^7b^	1.8 × 10^7bc^	ND
FS + CH + CC + TH14	6.5 × 10^8a^	ND	2.9 × 10^8a^	3.3 × 10^6d^	ND
SEM	0.436	–	0.071	0.052	–
**Material means**					
FS	2.2 × 10^8c^	–	2.2 × 10^7b^	1.4 × 10^8ab^	–
FS + CH	4.2 × 10^8a^	–	2.2 × 10^7b^	2.4 × 10^8a^	–
FS + CC	2.0 × 10^8c^	–	2.7 × 10^7b^	2.4 × 10^8a^	–
FS + CH + CC	3.4 × 10^8b^	–	1.5 × 10^8a^	1.1 × 10^8b^	–
**Inoculation means**					
Without TH14	7.2 × 10^7b^	–	3.0 × 10^7b^	2.7 × 10^7a^	–
With TH14	5.2 × 10^8a^	–	8.7 × 10^7a^	9.0 × 10^6b^	–
**P value**					
Materials (M)	< 0.001	–	< 0.001	0.015	–
Inoculation (I)	< 0.001	–	< 0.001	0.010	–
Interaction (M × I)	0.015	–	< 0.001	0.099	–

*FS* forage sorghum, *CH* Cavalcade hay, *CC* cassava chip, *TH14 L. casei* TH14 inoculant, *SEM* standard error of the means, *ND* not detected.

Means within columns with difference superscript letters differ at p < 0.05.

### Chemical composition and energy content of silage

The highest CP content (p < 0.05) was found in FS + CH, followed by FS + CH + CC, FS, and FS + CC silages (Table 4). The CP content was greater (p < 0.05) in LAB inoculated silage than that of control. The ADL contents were the highest (p < 0.05) in FS + CH silage. Silage prepared with LAB had a lower (p < 0.05) GE content than control. The FS and FS + CH silages had greater (p < 0.05) GE contents than the other two silages. The predicted ME (pME) contents of silage ranged from 9.21 to 10.58 MJ/kg DM (p < 0.05), with the superior values appearing in the order of FS + CC, FS + CH + CC, FS, and FS + CH silages, respectively. The pME contents of LAB-treated silage were greater (p < 0.05) than those of control. Interaction influenced (p < 0.05) the organic matter (OM), NDF, and ADF contents of silage.

**Table 4:** Chemical compositions and energy contents of the FS mixture silages.

Item	Chemical compositions (% DM)						GE (MJ/kg DM)	pME (MJ/kg DM)
	OM	CP	EE	NDF	ADF	ADL		
FS	96.98^a^	5.29^e^	2.36^b^	61.96^c^	39.59^a^	4.76^bc^	18.30^a^	9.62^d^
FS + TH14	96.83^a^	5.91^d^	2.72^a^	58.90^d^	35.13^c^	4.60^c^	18.28^a^	10.10^c^
FS + CH	95.08^cd^	9.77^a^	2.16^b^	70.49^a^	40.14^a^	5.67^a^	18.46^a^	9.21^e^
FS + CH + TH14	95.23^c^	9.94^a^	2.15^b^	66.06^b^	37.30^b^	5.51^a^	18.37^a^	9.54^d^
FS + CC	96.34^b^	4.74^f^	1.86^c^	59.65^d^	28.62^e^	2.82^d^	17.70^b^	10.49^ab^
FS + CC + TH14	96.23^b^	4.89^ef^	1.79^c^	52.19^e^	26.53^f^	2.53^d^	17.31^c^	10.58^a^
FS + CH + CC	95.08^d^	8.14^c^	1.71^c^	58.71^d^	32.32^d^	4.72^c^	17.74^b^	10.25^bc^
FS + CH + CC + TH14	94.99^d^	8.65^b^	1.72^c^	51.93^e^	31.44^d^	4.68^c^	17.62^b^	10.32^abc^
SEM	0.052	0.134	0.079	0.753	0.513	0.270	0.073	0.106
**Material means**								
FS	96.90^a^	5.60^c^	2.54^a^	60.43^b^	37.35^b^	4.68^b^	18.29^a^	9.86^c^
FS + CH	95.16^c^	9.86^a^	2.15^b^	68.28^a^	38.72^a^	5.59^a^	18.41^a^	9.38^d^
FS + CC	96.29^b^	4.82^d^	1.83^c^	55.92^c^	27.58^d^	2.68^c^	17.50^c^	10.54^a^
FS + CH + CC	95.03^d^	8.40^b^	1.71^c^	55.32^c^	31.88^c^	4.70^b^	17.68^b^	10.29^b^
**Inoculation means**								
Without TH14	95.87	6.99^b^	2.02	62.71^a^	35.17^a^	4.49	18.50^a^	9.89^b^
With TH14	95.82	7.35^a^	2.09	57.27^b^	32.60^b^	4.33	17.89^b^	10.14^a^
**P value**								
Materials (M)	< 0.001	< 0.001	< 0.001	< 0.001	< 0.001	0.001	< 0.001	< 0.001
Inoculation (I)	0.191	0.002	0.212	< 0.001	< 0.001	0.410	0.008	0.003
Interaction (M × I)	0.042	0.216	0.063	0.034	0.022	0.978	0.107	0.190

*FS* forage sorghum, *CH* Cavalcade hay, *CC* cassava chip, *TH14 L. casei* TH14 inoculant, *SEM* standard error of the means, *DM* dry matter, *OM* organic matter, *CP* crude protein, *EE* ether extract, *NDF* neutral detergent fiber, *ADF* acid detergent fiber, *ADL* acid detergent fiber, *GE* gross energy, *pME* predicted metabolizable energy.

Means within columns with difference superscript letters differ at p < 0.05.

**Figure 1:**
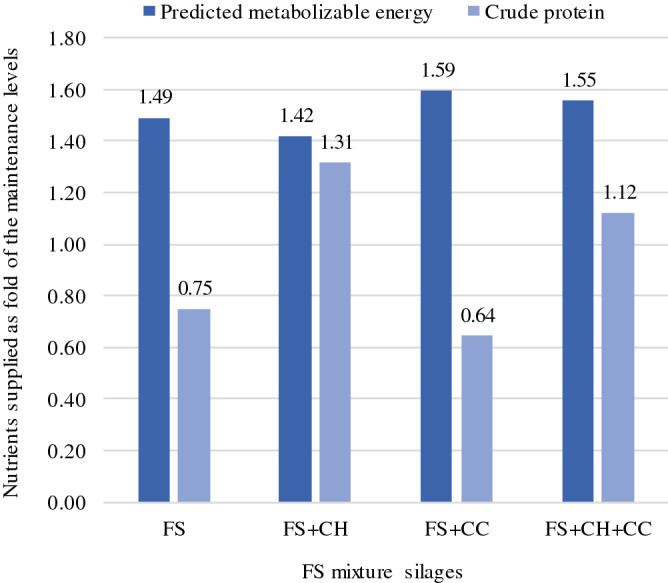
The nutritive values of the tested forage sorghum (FS) mixture silages estimated to supply the maintenance requirements of Zebu beef cattle in the growth stage according to current feeding standards^3^. *CH* Cavalcade hay (15% inclusion rate), *CC* dry cassava chip (10% inclusion rate).

Based on a synthesized comparison (Fig. 1), when compared to FS and FS + CC silages, the FS + CH and FS + CH + CC silages provided pME and CP contents above maintenance levels required by Zebu beef cattle in the growth stage.

### In vitro digestibility, total gas production, and methane production

After 24 h of incubation, the in vitro DM digestibility (IVDMD) in FS + CC and FS + CH + CC silages were higher (p < 0.05) than that in FS, and FS + CH silages (Table 5). The in vitro OM digestibility (IVOMD) was the highest (p < 0.05) in FS + CC silage, followed by FS + CH + CC, FS, and FS + CH silages. The total gas production and CH_4_ production (L/kg DM and % GE) were the highest (p < 0.05) in FS + CC silage. Compared to control silage, the LAB-treated silage increased IVDMD and IVOMD with decreased (p < 0.05) total gas and CH_4_ production. Interaction did not affect (p > 0.05) all parameters. When incubation was prolonged to 48 h, the effects of LAB inoculation were consistent with the findings at 24 h (Table 6). The FS + CC silage had greater (p < 0.05) IVDMD, IVOMD, total gas production, and CH_4_ production than the other silages.

**Table 5:** In vitro digestibility, total gas production and methane production of the FS mixture silages after 24-h incubation.

Item	IVDMD (%)	IVOMD (%)	TGP (L/kg DM)	Methane production			
				L/kg DM	L/kg IVDMD	L/kg IVOMD	% GE
FS	49.56^c^	52.67^c^	135.59^b^	16.84^bc^	34.04	33.02	3.64^bc^
FS + TH14	53.46^b^	56.90^b^	129.62^b^	16.15^bc^	30.18	29.29	3.49^bc^
FS + CH	46.63^c^	48.94^d^	133.67^b^	16.15^bc^	34.68	34.78	3.46^bc^
FS + CH + TH14	48.92^c^	52.27^c^	105.49^c^	14.48^c^	29.64	29.12	3.12^c^
FS + CC	57.96^a^	62.19^a^	166.72^a^	21.96^a^	38.14	36.80	4.91^a^
FS + CC + TH14	59.52^a^	63.28^a^	146.03^b^	17.97^b^	30.19	29.50	4.10^b^
FS + CH + CC	56.39^ab^	60.06^a^	140.18^b^	17.79^bc^	31.54	31.14	3.96^b^
FS + CH + CC + TH14	56.53^ab^	60.80^a^	135.39^b^	16.93^bc^	29.97	29.34	3.80^b^
SEM	1.157	1.055	7.059	1.037	2.257	2.171	2.250
**Material means**							
FS	51.51^b^	54.78^c^	132.61^bc^	16.49^b^	32.11	31.16	3.57^bc^
FS + CH	47.77^c^	50.61^d^	119.58^c^	15.32^b^	32.12	31.95	3.29^c^
FS + CC	58.74^a^	62.73^a^	156.37^a^	19.96^a^	34.17	33.16	4.51^a^
FS + CH + CC	56.46^a^	60.43^b^	137.80^b^	17.36^b^	30.76	30.25	3.88^b^
**Inoculation means**							
Without TH14	52.64^b^	55.96^b^	144.04^a^	18.18^a^	34.60^a^	33.94^a^	3.99^a^
With TH14	54.61^a^	58.31^a^	129.13^b^	16.38^b^	30.00^b^	29.32^b^	3.63^b^
**P value**							
Materials (M)	< 0.001	< 0.001	0.001	0.001	0.519	0.593	0.001
Inoculation (I)	0.024	0.004	0.006	0.022	0.008	0.006	0.032
Interaction (M × I)	0.448	0.299	0.294	0.376	0.566	0.622	0.444

*FS* forage sorghum, *CH* Cavalcade hay, *CC* cassava chip, *TH14 L. casei* TH14 inoculant, *SEM* standard error of the means, *IVDMD* in vitro dry matter digestibility, *IVOMD* in vitro organic matter digestibility, *TGP* total gas production, *GE* gross energy.

Means within columns with difference superscript letters differ at p < 0.05.

**Table 6:** In vitro digestibility, total gas production and methane production of the FS mixture silages after 48-h incubation.

Item	IVDMD (%)	IVOMD (%)	TGP (L/kg DM)	Methane production			
				L/kg DM	L/kg IVDMD	L/kg IVOMD	% GE
FS	59.40^e^	61.82^e^	171.54^c^	23.22^d^	39.10^bc^	38.75^c^	5.01^d^
FS + TH14	60.66^d^	63.17^d^	164.74^c^	22.32^d^	36.81^c^	36.51^c^	4.83^d^
FS + CH	52.83^f^	55.68^f^	149.32^d^	22.68^d^	42.94^ab^	42.84^ab^	4.86^d^
FS + CH + TH14	52.90^f^	56.25^f^	134.93^d^	19.86^e^	37.53^c^	37.10^c^	4.28^e^
FS + CC	67.38^a^	70.53^a^	204.70^a^	31.14^a^	46.23^a^	45.85^a^	6.96^a^
FS + CC + TH14	68.10^a^	71.14^a^	201.43^a^	30.25^ab^	44.42^a^	44.19^a^	6.91^a^
FS + CH + CC	63.03^c^	66.34^c^	186.11^b^	28.54^b^	45.28^a^	45.4^a^	6.36^b^
FS + CH + CC + TH14	64.78^b^	68.18^b^	169.31^c^	25.47^c^	39.31^bc^	39.33^bc^	5.72^c^
SEM	0.376	0.441	3.086	0.736	1.262	1.314	0.160
**Material means**							
FS	60.03^c^	62.49^c^	168.14^c^	22.77^c^	37.95^c^	37.63^c^	4.92^c^
FS + CH	52.86^d^	55.96^d^	142.13^d^	21.27^c^	40.23^bc^	39.97^bc^	4.57^d^
FS + CC	67.74^a^	70.83^a^	203.07^a^	30.70^a^	45.33^a^	45.02^a^	6.93^a^
FS + CH + CC	63.91^b^	67.26^b^	177.71^b^	27.01^b^	42.30^b^	42.29^b^	6.04^b^
**Inoculation means**							
Without TH14	60.66^b^	63.59^b^	177.92^a^	26.40^a^	43.39^a^	43.17^a^	5.80^a^
With TH14	61.61^a^	64.68^a^	167.60^b^	24.48^b^	39.52^b^	39.28^b^	5.43^b^
**P value**							
Materials (M)	< 0.001	< 0.001	< 0.001	< 0.01	< 0.001	< 0.001	< 0.001
Inoculation (I)	0.002	0.002	< 0.001	0.001	0.001	0.001	0.004
Interaction (M × I)	0.169	0.419	0.126	0.295	0.263	0.247	0.193

*FS* forage sorghum, *CH* Cavalcade hay, *CC* cassava chip, *TH14 L. casei* TH14 inoculant, *SEM* standard error of the means, *IVDMD* in vitro dry matter digestibility, *IVOMD* in vitro organic matter digestibility, *TGP* total gas production, *GE* gross energy.

Means within columns with difference superscript letters differ at p < 0.05.

## Discussion

The dynamic changes of microbial species and numbers during ensiling are important indicators that affect the fermentation quality and feed nutrient of silage. The abundance of epiphytic LAB of ≥ 10^5^ cfu/g FM is mostly preferred to promote lactic acid fermentation^9^. In this study (Table 1), the counts of LAB in FS, CH, and CC were lower than aerobic bacteria and yeast counts were. These results agreed with Cai et al.^5^, who indicated FS had low LAB counts (< 10^4^ cfu/g FM). This fact suggests the numbers of harmful bacteria should be controlled during silage fermentation by adding LAB inoculants^10^.

In this study, the addition of CH, CC, and CH + CC increased the DM content of FS mixture silage by 4 to 15% and could be an important factor that maintained its fermentation quality, as indicated by pH drops (< 3.9) and low butyric acid concentration (< 10 g/kg DM; Table 2). The results showed that the addition of CH increased silage pH value, which may be partly attributed to the influence of high buffering capacity in legume forage. Usually, the addition of legume CH increases the pH value of mixed silage. The TH14 displayed a higher lactic acid concentration and tended to have lower (p = 0.06) butyric acid concentrations than control. However, Khota et al.^11^ reported that strain TH14 could also increase acetic acid and propionic acid contents, and decrease butyric acid and NH_3_-N contents of FS silage. Understandably, natural bacteria present in FS may produce a different amount of short-chain fatty acids during ensiling.

The results showed that both LAB and aerobic bacteria counts were higher (p < 0.05) in FS + CH + CC + TH14 silages (Table 3). This interaction effect should associate with adding TH14, and some epiphytic LAB can survive in a low-pH condition of silage with different materials. This finding is contrary to the results of previous research; good quality silage generally reduces aerobic bacteria counts^11,12^. The reason for this is not very clear. Perhaps the aerobic spore-containing bacilli can grow in a relatively low pH environment such as silage. Future research needs to explore the relationship between spore forming aerobic bacteria and silage fermentation.

With an estimated DM intake of 2.3% body weight, Zebu beef cattle in the growth stage require about 75 g CP/kg DM and 6.62 MJ ME/kg DM^3^. Only FS + CH and FS + CH + CC silages could result in adequate levels needed to maintain healthy herds (Table 4). Although the sufficiency in ME supply is likely to exist in all FS mixture silages, both FS and FS + CC silages need to be supplemented with CP when used as feed (Fig. 1). Animals produced in these circumstances continually require protein levels above their maintenance requirement; otherwise, their body tissue degrades due to disease stemming from chronic malnutrition. In addition, such a practice emits extremely high-intensity CH_4_ due to negative production of beef cattle. Thus, our results strongly suggest considering protein supplements rather than NFC in FS. In this study, the NDF and ADF contents’ reducing effect on strain TH14 was influenced by interaction (p < 0.05; Table 4). Generally, LAB find it impossible to decompose plant fiber directly^9,11^. Perhaps other fiber-decomposing microbes that degrade fiber during silage fermentation could be found, which might lead to a reduction in fibrous contents.

The potential digestibility of forages for ruminants could be estimated with reasonable accuracy by using rumen fluid in vitro^13^. Therefore, in vitro tests have become important in qualifying whether to apply desirable treatments to in vivo experiments with animals. The results indicated FS + CC silage had consistently higher IVDMD, IVOMD, total gas production, and CH_4_ production than the other silages had (Tables 5 and 6). These findings largely associate with incorporated forage fiber in the NFC substrates of the CC portion, which lead to increased amounts of fermentable substrates in the in vitro rumen. The results agree with Chaudhry and Khan^14^, who reported that higher starch content in feeds could result in higher CH_4_ production than high fiber content could. Albores-Moreno et al.^15^ reported that the lower CH_4_ production could be due to the lower in vitro digestibility. Moreover, the addition of crude glycerol (a sugar alcohol) to a forage was recently reported to increase the in vitro CH_4_ production^16^. Given CC is ultimately important as a major energy feed source for ruminant production in these circumstances^3^, our results implied that it could be an attractive point for CH_4_-mitigation in this area.

The results showed TH14 decreased in vitro CH_4_ production with increased IVDMD and IVOMD compared to the control silage (Tables 5 and 6). We suspect the low pH value and the high lactic acid concentration of the LAB-treated silage may have led to a breakdown of the lignifications bound to the structural carbohydrates, which might be an action that improved the in vitro digestibility of FS mixture silage. The reduced methanogenesis due to LAB inoculation could stem from an abundance of ruminal lactic acid concentrations, which stimulates lactate-utilizing bacteria such as *Megasphaera elsdenii*, *Selenomonas ruminantium*, and *Veillonella parvula* to become hydrogen and CO_2_ sinks in the conversion of lactate to propionate^7^. In previous in vitro works, the effects of LAB additive on CH_4_ production were different among studies that mostly used different LAB species; probably because the LAB species have different modes of action to enhance rumen fermentation. Cao et al.^7^ found *L. plantarum* did not affect IVDMD of vegetable silages but did decrease the CH_4_ production. In addition, Ellis et al.^6^ suggested that the effects depended on the substrates and that most LAB inoculations were effective on grass silage but not corn silage, in addition to suggesting that some LAB strains can increase IVDMD with concurrent increased CH_4_ production.

In conclusion, the results suggest the addition of either CH or CH + CC makes this FS mixture silage type a promising candidate as an accessible dietary strategy for avoiding malnutrition in cattle. Understandably, when using FS or FS + CC, feeding cattle a concentrated protein supplement is strongly recommended. The addition of *L. casei* TH14 inoculant could help improve lactic acid production of these FS mixture silages and could modulate in vitro digestibility and in vitro enteric CH_4_ production. Determining the real CH_4_-mitigating potential of LAB additive in cattle feed requires evaluating the additive’s performance in vivo.

## Methods

The Animal Care and Use Committee of Khon Kaen University, Khon Kaen, Thailand, approved all experimental protocols. All methods were carried out in accordance with relevant guidelines and regulations.

### Experimental design and silage production

This experiment was conducted as a 4 × 2 factorial arrangement of treatments in a completely randomized design. In Factor A, the FS was prepared without or with CH 15% (FS + CH), CC 10% (FS + CC), and CH 15% + CC 10% (FS + CH + CC) on an FM basis. In Factor B, FS was left untreated (control) or was treated with *L. casei* TH14 at 1.0 × 10^5^ cfu/g FM. The aim of adding CH and CC was to balance the CP and ME contents, respectively, as recommended by Pholsen et al.^1^. The FS and CH were planted and harvested at 75 days on an experimental farm run by the Faculty of Agriculture, Khon Kaen University, Khon Kaen, Thailand. The CC was obtained from a local feedstuff supplier. The CH was prepared by wilting for 3 days in the field. The FS, CH, and CC were chopped separately at a theoretical length of 1 cm. Three replications of each treatment were prepared via plastic bags (100-g FM sample/bag) for the silage evaluations based on a small-scale silo technique according to previous studies^7,17^. Before packing and sealing, sample consisting of ensiling materials was prepared for each bag, which the LAB additive was added by spraying. The contents were mixed together. The silos were opened after 30 days of ensiling (23–32 °C) to enter a stable phase.

### Analytical procedures

The samples of materials and silages were analyzed for their microbial populations, including LAB, coliform bacteria, aerobic bacteria, yeast, and mold, using the plate count method as described by Kaewpila et al.^17^. The counts were reported in cfu/g FM. Silage fermentation products were analyzed from cold-water extracts^18^. Their pH was measured via a glass electrode. The NH_3_-N content was analyzed spectrophotometrically^19^. Acetic acid, propionic acid, butyric acid, and lactic acid concentrations were measured using high-performance liquid chromatography^18^.

Ensiling materials and silage samples were dried at 60 °C for 48 h, and then grinded (1 mm). The chemical composition was analyzed following the standard AOAC^20^ method, including DM, OM, CP, and ether extract (EE). The NDF and ADF were analyzed^21^ via a fiber analyzer (ANKOM 200, ANKOM Technology, NY, USA). The ADL was measured by solubilization with sulfuric acid^22^. The GE content was analyzed using an adiabatic bomb calorimeter (AC 500, LECO Corp., MI, USA). The NFC content of ensiling materials was calculated as the opposite fraction of ash, CP, EE, and NDF contents^23^. The pME content of silages was calculated using the equation by Thiputen and Sommart^24^:

$${\text{pME}}\; \, ({\text{MJ/kg}}\;{\text{DM}}) \, = \, 0.17 \, \times {\text{ EE}}\; \, (\% {\text{ DM}}) \, + \, 0.10 \, \times {\text{ IVOMD }}\;(\% ) \, + \, 3.952,$$

where IVOMD was obtained at 24 h of in vitro incubation.

An in vitro gas production technique was used following the method of Makkar et al.^25^. The cattle were fed a basal diet comprised of a 70:30 ratio of rice straw to concentrate on a DM basis. The concentrate ingredients were CC, rice bran, coconut kernel cake, palm kernel cake, urea, and a vitamin–mineral mixture at 500, 300, 110, 60, 10, and 20 g/kg, respectively, on a DM basis. Cattle were fed daily at 8:30 am and 4:00 pm. A stomach-tube sucker collected the rumen fluid before morning feeding from 3 heads of *Bos indicus* (181 ± 11.1 kg of body weight). Stomach tubing to obtain rumen fluid is a widely used alternative when such cannulated cattle are not available and does not invalidate the results of the comparison within this experiment. The rumen fluid was filtered through 4 layers of cheesecloth into prewarmed (39 °C) thermos bottles and transported immediately to the laboratory. Rumen fluid was diluted to 1:4 (v/v) with a buffer solution^25^. The serum bottles (50-mL capacity) containing ground silage sample (0.5 g) were closed via rubber stoppers and aluminum caps, injected with 40 mL of the rumen inoculums under CO_2_, and incubated at 39 °C in a water-bath checker (WNB22, Memmert GmbH + Co. KG, Schwabach, Germany). Incubations were in 2 batches for in vitro evaluations at 24 or 48 h of incubation with different flasks of rumen inoculum. Within each batch, 75 bottles were incubated, corresponding to 8 silage treatments × 3 silo replicates × 3 in vitro replicates + 3 blanks. The blanks were bottles consisting of only rumen inoculum. The gas produced was measured every 2 h using a 25-mL calibrated glass syringe. The piston was painted by graphite powder and petroleum jelly to ensure it was gastight. For each bottle, the gas measured in the glass syringe was stored in a gas bag (0.5-L capacity) via an air connector (1.68-mL capacity). After incubation, gas produced in a bottle’s headspace was purged into a gas bag by injecting 100-mL N_2_. The gas sample in each gas bag was analyzed for CH_4_ concentration (% v:v) via gas chromatography (GC8A, Shimadzu Corp., Kyoto, Japan). The CH_4_ production was calculated using the following equation:

$${\text{CH}}_{4} \;({\text{mL}}) \, = \, \left[ {{\text{TGP }}\;({\text{mL}}) \, + {\text{ AC}}\;({\text{mL}}) \, + {\text{ N}}_{2} \;({\text{mL}})} \right] \, \times \, \left[ {{\text{CH}}_{4} \;(\% \;{\text{v:v}})/100} \right],$$

where TGP is the total gas production, AC is the air connector volume (20.16 and 40.32 mL for 24 and 48 h of incubation, respectively), and N_2_ = 100 mL. The CH_4_ production was calculated as L/kg DM of substrate minus blanks and 39.54 kJ/L. The undigested sample was filtered through a glass filter crucible, washed by a pepsin solution, dried at 100 °C in a forced-air oven for 24 h, and weighed for IVDMD calculation. To measure IVOMD, the dried residues were burned in a muffle furnace at 550 °C for 3 h. Within each silage sample, 3 in vitro replications were screened to reveal the errors using a coefficient of variance criteria prior to averaging as the representative value.

### Statistical analysis

The data were analyzed for variance via the ANOVA procedure in SAS Version 6.12 (SAS Institute Inc., Cary, NC, USA) using the following model:

$${\text{Y}}_{{{\text{ijk}}}} = \, \mu \, + \, \alpha_{{\text{i}}} + \, \beta_{{\text{j}}} + \, \alpha \beta_{{{\text{ij}}}} + \, \varepsilon_{{{\text{ijk}}}} ,$$

where Y_ijk_ = observation, μ = overall mean, α_i_ = material effect (i = FS, FS + CH, FS + CC, and FS + CH + CC), β_j_ = LAB inoculation effect (j = without and with TH14), αβ_ij_ = the interaction effect (materials × LAB inoculation), and ε_ijk_ = error. The differences among treatment means were assessed by Duncan’s new multiple range test and the significance level was accepted at p < 0.05^26^.
