# Author Correction: Improving ensiling characteristics by adding lactic acid bacteria modifies in vitro digestibility and methane production of forage-sorghum mixture silage

**DOI:** 10.1038/s41598-021-87311-x

**Published:** 2021-04-07

**Authors:** Chatchai Kaewpila, Pongsatorn Gunun, Piyawit Kesorn, Sayan Subepang, Suwit Thip‑uten, Yimin Cai, Suradej Pholsen, Anusorn Cherdthong, Waroon Khota

**Affiliations:** 1 Faculty of Natural Resources, Rajamangala University of Technology Isan, Sakon Nakhon, 47160 Thailand; 2 Faculty of Liberal Arts and Sciences, Sisaket Rajabhat University, Sisaket, 33000 Thailand; 3 Faculty of Agricultural Technology, Sakon Nakhon Rajabhat University, Sakon Nakhon, 47000 Thailand; 4 Japan International Research Center for Agricultural Sciences (JIRCAS), Tsukuba, Ibaraki 305‑8686 Japan; 5 Faculty of Agriculture, Khon Kaen University, Khon Kaen, 40002 Thailand

Correction to: *Scientific Reports* https://doi.org/10.1038/s41598-021-81505-z, published online 21 January 2021

The Acknowledgements section in the original version of this Article was incomplete.

“This study was funded by the National Research Council of Thailand (NRCT, Funding no. 572103). The authors thank Khon Kaen University, Japan International Research Center for Agricultural Sciences (Japan), Rajamangala University of Technology Isan, Sisaket Rajabhat University, and Sakon Nakhon Rajabhat University for the infrastructure and laboratory facilities.”

now reads:

“This study was funded by the National Research Council of Thailand (NRCT, Funding no. 572103) and the Increase Production Efficiency and Meat Quality of Native Beef and Buffalo Research Group, Department of Animal Science, Faculty of Agriculture, Khon Kaen University. The authors thank Rajamangala University of Technology Isan, Sisaket Rajabhat University, Sakon Nakhon Rajabhat University, and Japan International Research Center for Agricultural Sciences (Japan) for the infrastructure and laboratory facilities.”

The original Article has been corrected.

